# Editorial: Advances in pea breeding and genomics

**DOI:** 10.3389/fpls.2024.1430421

**Published:** 2024-06-03

**Authors:** Thomas D. Warkentin, Petr Smýkal, Pei Xu, Kevin McPhee

**Affiliations:** ^1^ Crop Development Centre/Department of Plant Sciences, University of Saskatchewan, Saskatoon, SK, Canada; ^2^ Department of Botany, Faculty of Sciences, Palacký University, Olomouc, Czechia; ^3^ Key Laboratory of Specialty Agri-Product Quality and Hazard Controlling Technology of Zhejiang, College of Life Sciences, China Jiliang University, Hangzhou, China; ^4^ Department of Plant Science and Plant Pathology, Montana State University, Bozeman, MT, United States

**Keywords:** Pea, Pisum, breeding, genomics, QTL

Pea is an important food legume crop in temperate regions of the world. World dry pea production averaged 13.5 million tons/year (2013–2022), while vegetable pea production averaged 19.2 million tons/year over that period (FAOSTAT). Dry pea production has been relatively stable while vegetable pea production has been increasing. The leading producers of dry pea are Canada, Russia, China, and India, while the leading producers of vegetable pea are China and India. Dry pea is used for whole seeds, dehulled seeds, flour, and in fractionated forms including protein concentrates and isolates and starch concentrates and isolates (Warkentin et al., 2015). Pea has become a leading crop type in the growing plant-based protein industries. Vegetable pea is used in the fresh form as shelled peas, snap peas, and snow peas. As a nitrogen-fixing grain legume crop, pea fits well into cereal-based crop rotations (Rubiales et al., 2019). As pea production typically does not require nitrogen fertilizer, it is a highly beneficial cropping option for addressing the climate change objective of reducing greenhouse gas emissions.

This Research Topic, ‘*Advances in pea breeding and genomics*’, includes five papers that address important aspects of pea production and utilization. Weeden et al. explored genetic diversity in *Pisum fulvum* L., a key wild relative of *Pisum sativum* L., the cultivated pea. Their study of 90 P*. fulvum* accessions showed remarkable sequence diversity at the *STAYGREEN (SGR)* locus encoding Mendel’s cotyledon color gene. Fifty-seven alleles were identified based on the sequence of the third intron of *SGR*. The *SGR* genotype was more effective than morphological traits in distinguishing the accessions. The accessions were classified as Group A (mainly northern Israel) and Group B (mainly the arid regions of southern Israel). *P. fulvum* accessions have been explored by breeders, potentially as donors of adaptive or disease resistance traits; for example, improved resistance to the scochyta complex (Jha et al., 2016, Jha et al, 2017) and drought tolerance (Naim-Feil et al., 2017) was identified in several *P. fulvum* accessions.

Three of the papers in this Research Topic described progress in mapping key traits of interest in pea breeding. Yan et al. reported on the discovery of a key QTL associated with resistance to two bruchid (*Callosobruchus*) species. Bruchid beetles can cause substantial damage to pea and faba bean during the cropping season and especially in storage in warmer regions of the world. Nearly 30 years ago, Hardie et al. (1995) reported on several sources of resistance to bruchid in pea, but few authors have reported on the genetic control of resistance. Here, Yan et al. reported on a major QTL, explaining greater than 50% of the variation for resistance to *C. chinensis* L. and *C. maculatus* Fab in the population. By fine mapping, they narrowed the key genomic region associated with resistance down to 1.07 Mb on pea chromosome 2. They proposed that a xylanase inhibitor-encoding gene in this region was the candidate gene for bruchid resistance. A marker associated with this allele could be useful in pea breeding.

Delvento et al. used QTL mapping to identify key genomic regions in pea associated with resistance to crenate broomrape (*Orobanche crenata* Forsk.), an important parasitic weed in grain legume production regions near the Mediterranean. An F_7_ recombinant inbred line (RIL) population of 148 lines arising from the cross ROR12 (resistant) X Sprinter (susceptible) was evaluated in field trials over two years. Mapping revealed three QTLs associated with resistance to broomrape. KASP assays for use in pea breeding were developed from the key marker within each QTL.

Moving from field traits to seed quality traits, Gali et al. used QTL analysis to identify key genomic regions associated with seed protein concentration in pea in two RIL populations. Pea varieties with increased protein concentration will be attractive to companies fractionating peas for plant-based protein applications. RIL population arising from the cross MP 1918 X P0540–91 (called PR-30) was developed by Agriculture and Agri-Food Canada, while the RIL population arising from Ballet X Cameor (called PR-31) was developed by INRAe, France. These RIL populations were phenotyped in multi-location field trials over two years in western Canada. Three QTLs associated with pea seed protein concentration were identified in PR-30 and five from PR-31. Most of these QTLs were different from previously reported QTLs for pea seed protein concentration.

Finally, Crosta et al. utilized genomic prediction for agronomic traits in a pea germplasm collection. Their goal was to use genomic prediction for polygenic traits to identify promising germplasm accessions which might be used in pea breeding. They evaluated 220 pea landraces or old cultivars, as well as 11 modern cultivars, using a panel of 41,000 single nucleotide polymorphism (SNP) markers. Several regions on chromosome 6 were found to be associated with vegetative and reproductive organ pigmentation. Several SNPs were associated with grain yield and straw yield. Genomic prediction models had moderately high predictive ability for most key quantitative traits of interest to pea breeders.

Overall, this Research Topic on *Advances in pea breeding and genomics* should prove valuable for pea breeders and researchers. Additionally, these studies should inform research in related legume crops.

## Author contributions

TW: Writing – review & editing, Writing – original draft. PS: Writing – review & editing. PX: Writing – review & editing. KM: Writing – review & editing.
